# 

**Published:** 1865-11

**Authors:** 


### Books and Pamphlets Received.

The Practice of Medicine and Surgery applied to the Diseases and Accidents incident to Women. By William H. Byford, A. M., M. D. Philadelphia: Lindsay & Blakiston. 1865.

Materia Medica, for the Use of Students. By John B. Biddle, M. D. With Illustrations. Philadelphia: Lindsay & Blakiston. 1865.

The Use of the Larynoscope in Diseases of the Throat. With an Appendix on Rhinoscopy. By Morell Mackenzie, M. D., Lond., M. R. C. P. Philadelphia: Lindsay & Blakiston. 1865.

Lectures on the Diseases of the Stomach, with an Introduction on its Anatomy and Physiology. By William Brinton, M. D., F. R. S. From the second English edition. Philadelphia: Lea & Blanchard. 1865.

Stimulants and Narcotics, their Mutual Relations: With Special Researches on the Action of Alcohol, Æther and Chloroform on the Vital Organism. By Francis E. Anstie, M. D., M. R. C. P. Philadelphia: Lindsay & Blakiston. 1865.

The Student's Book of Cutaneous Medicine and Diseases of the Skin. By Erasmus Wilson, F. R, S. New York: William Wood & Co. 1865.

The American Journal of Insanity. Edited by the Medical Officers of the New York State Lunatic Asylum. October, 1865.

Specialties in Medicine. By Henry D. Noyes, M. D. Read before the American Ophthalmological Society, June, 1865.

Catalogue of the Trustees, Overseers, Faculty and Students of the Berkshire Medical College, for the year 1865. Pittsfield, Mass., October, 1865.

Report on the Use of Pressure in the Treatment of Gonorrhœal and Purulent Ophthalmia. By Surgeon Joseph S. Hildreth, U. S. V. New York: John Medole. 1865.

American Educational Monthly. Devoted to Popular Instuction and Literature. September, 1865.

Report of the Willard Asylum, and Provision for the Insane. 1865.

Transactions of the American Ophthalmological Society. Second Annual Meeting. New York, June, 1865.

Reports of the Trustees and Superintendent of the Tennessee Hospital for the Insane, presented to the General Assembly, April 3, 1865.

A Report upon the Epidemic occurring at Maplewood Young Ladies' Institute, Pittsfield, Mass., in July and August, 1864: Including a Discussion of the Causes of Typhoid Fever. By A. B. Palmer, M. D., C. L. Ford, M. D., and Pliny Earle, M. D.

